# Accumulation of Platinum Nanoparticles by *Sinapis alba* and *Lepidium sativum* Plants

**DOI:** 10.1007/s11270-015-2381-y

**Published:** 2015-04-01

**Authors:** Monika Asztemborska, Romuald Steborowski, Joanna Kowalska, Grazyna Bystrzejewska-Piotrowska

**Affiliations:** 1 Isotope Laboratory, Faculty of Biology, University of Warsaw, Miecznikowa 1, 02-096 Warsaw, Poland; 2 Faculty of Chemistry, University of Warsaw, Pasteura 1, 02-093 Warsaw, Poland

**Keywords:** Platinum, Nanoparticles, Accumulation, Plants, *Sinapis alba*, *Lepidium sativum*

## Abstract

Nanoparticles (NPs) are commonly used, and concerns about their possible adverse effects are being voiced as well. However, little is known about the fates of NPs released to the environment. The aim of the study was to (i) evaluate the ability of *Sinapis alba* and *Lepidium sativum* plants to take up platinum nanoparticles (Pt-NPs) and translocate them to aboveground organs, (ii) compare the accumulation efficiency of different forms of platinum and (iii) identify the forms in which platinum is stored in plant tissues. Plants were cultivated on medium supplemented with different concentrations of Pt-NPs and [Pt(NH_3_)_4_](NO_3_)_2_. Platinum content in plants was determined using inductively coupled plasma mass spectrometry. For the identification of the presence of Pt-NPs in plant tissues, gamma spectrometry following iron irradiation was applied. It was found that *L. sativum* and *S. alba* are tolerant to applied concentrations of Pt-NPs and have an ability to take up platinum from the medium and translocate it to aboveground organs. The highest concentration of platinum was observed in plant roots (reaching 8.7 g kg^−1^ for *S. alba*). We tentatively conclude that platinum is accumulated as nanoparticles. The obtained results suggest future application of plants for phytoremediation and recovery of noble metal nanoparticles.

## Introduction

The unique properties of nanoscale materials, associated with their high surface-to-volume ratio, have caused a rapid increase in the implementation of nanotechnologies in the last 20 years. Many products based on nanotechnologies are in everyday use now and many more new ones are expected to appear on the market soon (Maynard et al. 2006; Rejeski and Lekas 2008). Nanoparticles of platinum (Pt-NPs) are of great scientific interest as they have many industrial and biomedical applications. Platinum is a rare element used in jewellery and as a catalyst. In macro-size form, platinum is not only one of the most effective but also very expensive catalyst. As the catalytic reactivity depends on the size and shape of particles, the effectiveness of catalytic processes on nanoparticles is much higher because the reactive surface area increases significantly in comparison with microparticles. Platinum nanoparticles have also been used in biomedical applications (Bhattacharya and Murkherjee 2008) and nanocrystals of FePt@CoS_2_ have been found to be more potent in killing HeLa cells than cis-platinum (Gao et al. 2007). Nanoscale platinum is suitable for designing new electrochemical sensors and biosensors (Luo et al. 2006). The wide range of applications of Pt-NPs creates a growing demand for more efficient and cost-effective processes for their synthesis. Platinum nanoparticles can be made, e.g. by reduction of hexachloroplatinate (Devi and Rao 2000). Biological methods for nanoparticle synthesis using microorganisms, plants or plant extracts are very attractive and eco-friendly alternative to chemical and physical methods (Konishi et al. 2007; Mohanpuria et al. 2008; Kaushik et al. 2010; Song et al. 2010).

One of the side effects of the growing application of nanotechnologies is the release of nanomaterials to the environment and the creation of a new type of waste, containing residue nanomaterials—nanowaste (Bystrzejewska-Piotrowska et al. 2009). This calls for a comprehensive investigation of the uptake, bioaccumulation and biotransformation of nanomaterials and the risks associated with their use. Efficient removal of NPs from the contaminated environment may prevent their ecotoxicity (Oberdorster et al. 2005). Additionally, recovery of nanoparticles of noble metals from the contaminated wastewater, water or soil is economically justified. Here, phytoremediation techniques seem promising as they enable both decontamination of the environment and recovery of the nanoparticles, being simultaneously eco-friendly and relatively cost-effective (Kidd et al. 2009; Paz-Alberto and Sigua 2013). Cost estimates indicate savings for a phytoremediation compared to a conventional treatment to be 50 to 80 % depending on contaminant, matrix and applied remediation techniques (Environmental Protection Agency 2000, EPA/600/R-99/107).

The ability of platinum uptake by hydroponically cultivated plants—Indian mustard (*Sinapis alba* L.) and Anawa maize (*Zea mays* L.)—was investigated by Kowalska et al. (2004a, b) and Hawienczyk et al. (2005). It was found that both studied plant species poses tolerance to relatively high inorganic Pt concentration (up to 500 mg Pt L^−1^ in a form of [Pt(NH_3_)_4_](NO_3_)_2_) and efficiently uptake and transport platinum to aboveground parts. The platinum content: exciding 400 and 200 mg kg^−1^ for roots of Indian mustard and Anawa maize, respectively, and about 40 and 10 mg kg^−1^ for aboveground organs of both species were found. It is known that some plant species are hyperaccumulators of platinum, but information on the bioaccumulation of Pt nanoparticles is lacking. Zhu et al. (2008) showed that iron oxide nanoparticles (Fe_3_O_4_) were taken up by *Cucurbita maxima* roots and translocated through the plant tissues while no uptake or transport of iron oxide nanoparticles was observed for *Phaseolus limensis*. We reported accumulation of iron oxide nanoparticles by *Lepidium sativum* (Bystrzejewska-Piotrowska et al. 2012). Lin et al. (2009) found that C70 fullerene not only could be easily taken up by the roots of *Oryza sativa* and transported to shoots but also could be transported downward from leaves to roots through the phloem if it entered the plants through the leaves. Other studies indicated no upward translocation of ZnO nanoparticles from *Lolium perenne* roots to shoots (Lin and Xing 2008). For metallic nanoparticles, Cu nanoparticles could be taken up and accumulated by bean and wheat plants (Lee et al. 2008).

The aim of the present study was to evaluate the ability of *S. alba* and *L. sativum* plants to take up platinum nanoparticles and translocate them to above ground organs. In our previous studies, we have shown that *S. alba* accumulates platinum (Kowalska et al. 2004a, b; Hawienczyk et al. 2005) while *L. sativum* can accumulate iron oxide nanoparticles (Bystrzejewska-Piotrowska et al. 2012). Additionally, we compared the accumulation efficiency of different forms of platinum and attempted to identify the forms in which it is stored in plant tissues.

## Materials and Methods

Platinum nanoparticles (Pt-NPs, nanopowder, particle size <50 nm (TEM), spec. surface area BET 98 m^2^/g) were purchased from Sigma-Aldrich. According to the Material Safety Data Sheet, platinum nanoparticles may be harmful if inhaled (causing respiratory tract irritation), absorbed through skin (skin irritation) and may cause eye irritation. Safety glasses, gloves, protective clothing and air-purifying respirators were used during handling of platinum nanoparticles.

The particle size and morphology were assessed using LEO 912AB transmission electron microscope (Zeiss) equipped with a Proscan High Speed Slow Scan CCD camera. The TEM analysis was performed using 20 mg L^−1^ water suspensions. The platinum complex [Pt(NH_3_)_4_](NO_3_)_2_ was from Sigma-Aldrich. The chemicals used for preparation of plant nutrient solutions were from Avantor Performance Materials Poland S.A., Poland. All solutions were prepared in 18 MΩ cm Milli-Q water (Millipore, USA). All solutions of nanoparticles were sonicated for 30 min.

For irradiation, samples of Pt nanoparticles (80–100 mg) were weighed directly into HDPE snap-cap capsules (Faculteit Biologie, Vrije Universiteit, Amsterdam), wrapped in aluminium foil and irradiated at the MARIA nuclear reactor (Świerk, Poland) for 10 min at a thermal neutron flux of 10^14^ cm^−2^ s^−1^. After 10 days of cooling, the samples were unwrapped and used for plant cultivation. The purity of irradiated nanoparticles was confirmed using a GENIE-2000 gamma-ray spectrometer (Canberra Gamma Spectrometry System) with an HPGe detector (Canberra), active volume 255 cm^3^, well type, well diameter 16 mm and depth 40 mm, resolution 2.4 keV for the 1,332.4 keV peak of ^60^Co and relative efficiency 24 %.

*L. sativum* plants were cultivated for 3 days in 300-mL containers (50 plants/container) with distilled water. After that time, the plants were transferred to the growth medium supplemented with Pt nanoparticles at concentrations 0 (control), 1, 10 or 100 mg L^−1^. Cultivation was carried out for the next 5 days. In experiments with irradiated Pt-NPs (final concentration 85 mg L^−1^), the cultivation was 2 days only due to the short half-life time of the isotopes.

*S. alba* L. was cultivated in 1.5-L containers (20 plants/container) according to the procedure described elsewhere (Kowalska et al. 2004b). In the first experiment, the nutrient solution was supplemented with Pt-NPs at 0 (control), 1, 10 or 50 mg L^−1^. The second experiment cultivation was performed in two variants. Platinum was added to the nutrient solution to a final concentration of 1 mg L^−1^ as inorganic salt or as nanoparticles. *S. alba* plants were cultivated for 3 weeks.

After cultivation as above, the plants were harvested, roots were washed with deionized water and plants were divided into roots and shoots, which were than dried and ground.

For analysis of platinum content, plant samples were acid-digested using ETHOS 1 microwave laboratory system with an ATC-400-CE automatic temperature control (Milestone, Italy). About 250 mg of dried sample was weighed into a PTFE vessel and 3 mL of aqua regia was added (65 % HNO_3_ and 37 % HCl, both from Merck). After digestion, samples were transferred quantitatively into volumetric flasks and brought up to 25 mL with water. Samples were analyzed by inductively coupled plasma mass spectrometry ICP MS (ELAN 6000 ICP mass spectrometer (PE-SCIEX, Concord, Canada)).

The radionuclide content (energy line 81.00 keV) was determined in dried plant samples by means of a gamma spectrometer with an HPGe detector (Canberra Packard).

## Results and Discussions

**Fig. 1:**
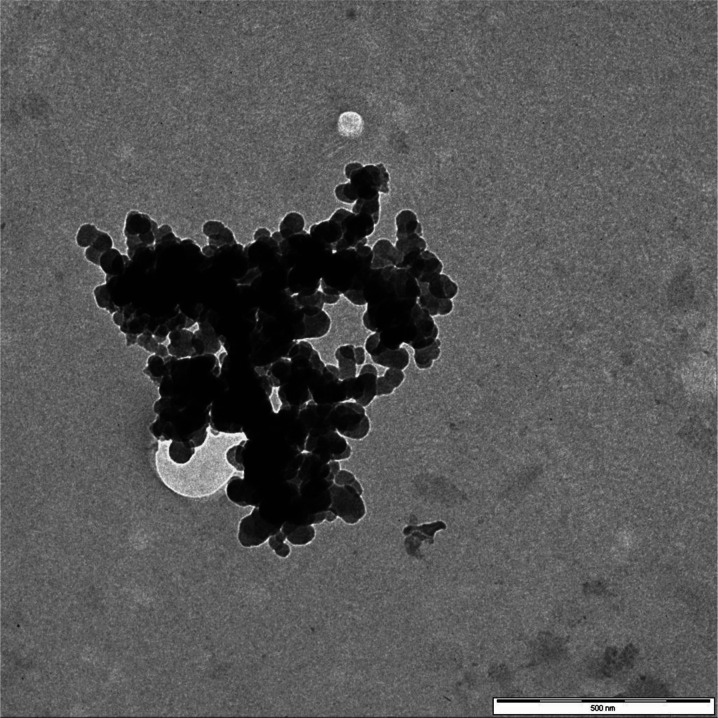
Transmission electron micrograph of Pt nanoparticles (20 mg L^−1^ suspension in water). Scale bar 500 nm

Before uptake experiments, we characterize the platinum nanoparticles in standard solution using transmission electron microscopy (Fig. 1). Most particles were spherical, and their size did not exceed 50 nm in agreement with the declaration of the supplier.

The *L. sativum* and *S. alba* plants appeared tolerant to the applied relatively high concentrations of Pt-NPs because no visible phytotoxic effects were observed. The colour of the plants, biomass production, root system or tissues hydration did not differ substantially between the control and the Pt-NP-exposed plants. However, detailed tests for possible phytotoxic effects on the cellular level were not undertaken.

Both plant species investigated took up platinum NPs from the medium in considerable amounts. It was found in both roots and shoots, although at markedly different concentrations (Table 1). In both species, the roots contained significantly higher concentration (per dry weight) of platinum than shoots.

**Table 1:** Pt content and transfer factors for *Lepidium sativum* and *Sinapis alba*. Values given are mean ± SD; *n* ≥ 3

Pt-NPs concentration in medium (mg L^−1^)	Pt content in plants (mg kg^−1^) (transfer factor)			
	*Lepidium sativum*		*Sinapis alba*	
	Shoots	Roots	Shoots	Roots
1	3.1 ± 0.3 (3.1)	148 ± 12 (148)	3.5 ± 0.3 (3.5)	190 ± 15 (190)
10	17.6 ± 1.1 (1.8)	179 ± 16 (17.9)	16.5 ± 1.0 (1.7)	1,855 ± 166 (185)
100/50^a^	563 ± 33 (0.5)	7,460 ± 516 (7.5)	54 ± 3 (1.1)	8,752 ± 605 (175)
85^b^	3.9 ± 0.4 (0.04)	278 ± 33 (3.3)	–	–

*TF* platinum concentration in plants [mg kg^−1^ dry weight] divided by that in the growth medium [mg L^−1^]

^a^100 mg L^−1^ for *L. sativum* and 50 mg L^−1^ for *S. alba*

^b^Cultivation time 48 h; irradiated Pt-NPs

*L. sativum* exposed to the highest Pt-NPs concentration applied (100 mg L^−1^) accumulated almost 7.5 g kg^−1^ of platinum in roots. In plants grown on the medium with Pt-NPs at 1 or 10 mg L^−1^, the amount of platinum in roots was about 50 and 40 times lower, respectively. The platinum content in shoots clearly depended on the Pt-NP content in medium, and for the highest platinum concentration applied, it reached nearly 0.6 g kg^−1^. The efficiency of platinum accumulation, defined as transfer factor (TF; platinum concentration in plants [mg kg^−1^ dry weight] divided by that in growth medium [mg L^−1^]) decreased with the increase of particle concentration, both for shoots and roots of *L. sativum*.

The accumulation of platinum in shoots of *S. alba* exposed to Pt-NPs at 1 or 10 mg L^−1^ was similar for *L. sativum* cultivated in the same media, while for the highest concentration of platinum nanoparticles used, it was about 10 times lower than that in *L. sativum*. The platinum content in roots of *S. alba* was significantly higher in comparison with *L. sativum*. The TF decreased with the increase of NP concentration for shoots, but was constant for roots.

**Fig. 2:**
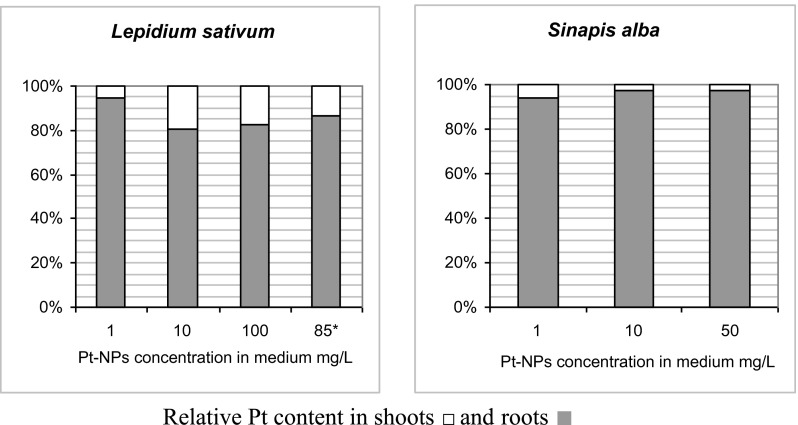
Distribution of platinum between roots and shoots of *Lepidium sativum* and *Sinapis alba*

At the lowest concentration of Pt-NPs in the growth medium, 1 mg L^−1^, 95 % of the NPs taken up by *L. sativum* accumulated in roots and only 5 % in the shoot (Fig. 2). At the highest concentration, the fraction of platinum translocated to the shoot increased significantly to ca. 20 %. *S. alba* accumulated Pt mainly in roots (∼95 %) as well, regardless of the Pt-NPs concentration used. A comparison of the results for the two plant species shows that they differ substantially at the efficiency and mode of Pt-NPs uptake and transport. Similar species specificity was reported earlier for a number of plant species and diverse metals (Lee et al. 2008; Lin and Xing 2008; Zhu et al. 2008; Lin et al. 2009; Bystrzejewska-Piotrowska et al. 2012).

**Fig. 3:**
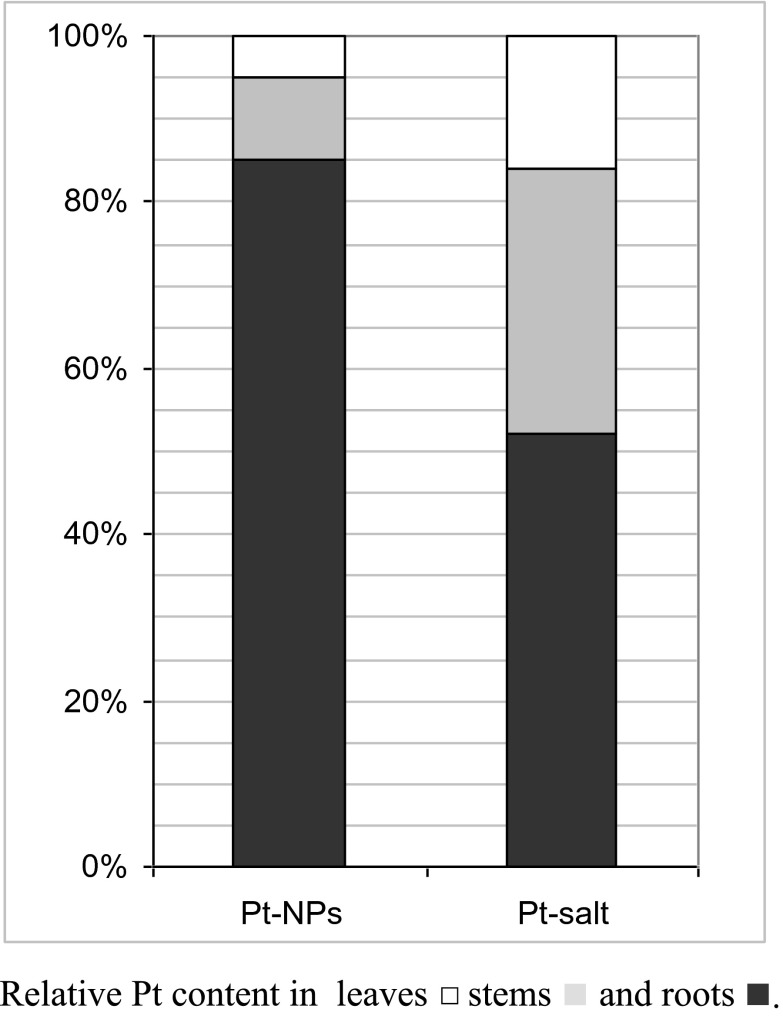
Distribution of platinum between roots and shoots of *Sinapis alba* exposed to platinum in the form of [Pt(NH_3_)_4_](NO_3_)_2_ or Pt-NPs. Platinum concentration in medium was in both case 1 mg L^−1^

Further experiments focused on efficiency of the platinum accumulation depending on its form in the growth medium. *S. alba* was exposed to 1 mg L^−1^ of platinum in the form of nanoparticles or an inorganic water soluble salt. As expected, in plants exposed to the salt, the amounts of platinum were about 220, 30 and 3 times higher for leaves, stems and roots, respectively, than in plants exposed to nanoparticles (Table 2). *S. alba* cultivated on the Pt salt-contaminated medium accumulated more than 60 % of platinum in shoots, while in plants grown on medium with NPs, only 15 % of platinum was transported to aboveground organs (Fig. 3). These results confirm a better bioavailability of metals from a salt than from nanoparticles. However, it must be stressed that the platinum transport from the medium to shoots is much more effective for the salt, while the accumulation in roots is only twice higher. The rather high amount of platinum found in roots exposed to Pt-NPs and its poor upward transport are partly caused by adsorption of the nanoparticles on the root surface. The adsorption of platinum nanoparticles on the root surface was macroscopic—visible without the use of any equipment.

**Table 2:** Pt content and transfer factors for *Sinapis alba* exposed to platinum in the form of [Pt(NH_3_)_4_](NO_3_)_2_ or Pt-NPs. Platinum concentration in medium was in both case 1 mg L^−1^. Values given are mean ± SD; *n* ≥ 3

	Pt content in plants (mg kg^−1^) (transfer factor)	
	Pt(NH_3_)_4_](NO_3_)_2_	Pt-NPs
Leaves	120 ± 4 (120)	0.55 ± 0.20 (0.6)
Stems	215 ± 6 (215)	6.8 ± 4.3 (6.8)
Roots	384 ± 4 (384)	145 ± 14 (145)

*TF* platinum concentration in plants [mg kg^−1^ dry weight] divided by that in the growth medium [mg L^−1^]

Relatively high amount of platinum in plants exposed to nanoparticles does not answer the question whether the platinum is taken up as nanoparticles. During further studies, we sought the presence of platinum ions in the growth medium and plants. For this purpose, growth media (kept under experimental conditions with or without plants) and water plant extracts were filtered and ultracentrifuged, and the amount of platinum in the supernatant was determined. It was below the detection limit, so no or very little Pt ions originating from nanoparticles are present in the medium or in the plants.

The problem of nanoparticle accumulation by plants is very complex because in plant cells, many different processes can take place. For instance, taking into consideration that some plants can reduce metal ions to elemental NPs inside their tissues (Harris and Bali 2008; Bali et al. 2010), we can expect that even if platinum was taken up as ions, it could be present as NPs in plant cells. In short, nanoparticles accumulated in plants can potentially come from two sources—direct accumulation of nanoparticles and reduction of metal ions taken up.

As an excellent tool for tracing the environmental fate of NPs and their uptake and accumulation in organisms (Oughton et al. 2008; Bystrzejewska-Piotrowska et al. 2012), gamma spectrometry following neutron activation of platinum was used for Pt-NP analysis in plant tissues. Details of the procedure are described elsewhere (Bystrzejewska-Piotrowska et al. 2012). This method was previously successfully applied for analysis of accumulation of Fe_3_O_4_ nanoparticles by *L. sativum* (Bystrzejewska-Piotrowska et al. 2012). The activity measured in the plant samples was within the range 1.2 kBq g^−1^ (shoots)–426.5 kBq g^−1^ (roots), so we could expect that Pt nanoparticles were present in plants. The amount of platinum in *L. sativum* after only 2 days of cultivation was about 4 and 280 mg kg^−1^ for shoots and rots, respectively, showing that the NPs are vigorously taken up by plants from the first days of cultivation.

On the basis of the obtained results, some general conclusions can be drawn. Both plant species can be potentially used for phytoremediation of the environment contaminated with platinum nanoparticles. The higher biomass production makes *S. alba* better for that purpose. Plants can also be used to recover nanoparticles from the environment. It is especially important in the case of platinum—a very useful and a very expensive metal. We can also conclude that, as for the question of environment contamination is concerned, nanoparticles are much safer than inorganic forms of platinum are. Accumulation in aboveground organs is 200 times less for NPs than for platinum ions. Although appropriate experiments were not performed here, one can safely assume that the low accumulation of platinum nanoparticles in the stem results in a negligible metal accumulation in seeds. In conclusion, the chances of NPs entering the food chain are much lower in comparison with ionic platinum.

Finally, it must be stressed that plants have an ability to reduce platinum ions to form nanoparticles (Song et al. 2010; Bali et al. 2010). Plant extracts are used for Pt-NP synthesis as a very promising and ecologically safe procedure. It is likely that ionic platinum that is taken up can be transformed in plant cells to metallic platinum in the form of individual nanoparticles. The conditions in plant tissues favour accumulation of nanoparticles.

During the presented studies, it was found that *L. sativum* and *S. alba* are tolerant to relatively high concentrations of Pt-NPs and have an ability to take up platinum from the medium and translocate it to aboveground organs. However, in both of these species, ca. 90 % remains associated with the roots. The efficiency of platinum accumulation depends on its form and is different for the two plant species. We propose that platinum is accumulated as nanoparticles. The obtained results give a perspective for future application of plants for environment phytoremediation and recovery of noble metal nanoparticles.

## References

[CR1] Bali R, Siegele R, Harriset AT (2010). Biogenic Pt uptake and nanoparticle formation in *Medicago sativa* and *Brassica juncea*. Journal of Nanoparticle Research.

[CR2] Bhattacharya R, Murkherjee P (2008). Biological properties of “naked” metal nanoparticles. Advanced Drug Delivery Reviews.

[CR3] Bystrzejewska-Piotrowska G, Golimowski J, Urban L (2009). Nanoparticles: their potential toxicity, waste and environmental management. Waste Management.

[CR4] Bystrzejewska-Piotrowska G, Asztemborska M, Steborowski R, Ryniewicz J, Polkowska-Motrenko H, Danko B (2012). Application of neutron activaton for investigation of Fe_3_O_4_ nanoparticles accumulation by plants. Nukleonika.

[CR5] Devi GS, Rao VJ (2000). Room temperature synthesis of colloidal platinum nanoparticles. Bulletin of Materials Science.

[CR6] Environmental Protection Agency, USA, (2000) Introduction to phytoremediation, EPA/600/R-99/10, 2000.

[CR7] Gao J, Liang G, Zhang B, Kuang Y, Zhang X, Xu B (2007). FePt@CoS2 yolk-shell nanocrystals as a potent agent to kill HeLa cells. Journal of the American Chemical Society.

[CR8] Hawienczyk M, Bystrzejewska-Piotrowska G, Kowalska J, Asztemborska M (2005). Platinum bioaccumulation by mustard plants (*Sinapis alba* L.). Nukleonika.

[CR9] Harris, A. T., & Bali, R. (2008). On the formation and extent of uptake of silver nanoparticles by live plants. *Journal of Nanoparticles Research, 10, *691–695.

[CR10] Kaushik N, Thakkar MS, Snehit S, Mhatre MS, Rasesh Y, Parikh MS (2010). Biological synthesis of metallic nanoparticles. Nanomedicine: Nanotechnology, Biology and Medicine.

[CR11] Kidd P, Barcelo J, Bernal MP, Navari-Izzo F, Poschenrieder C, Shilev S, Clementec R, Monterroso C (2009). Trace element behaviour at the root–soil interface: implications in phytoremediation. Environmental and Experimental Botany.

[CR12] Konishi Y, Ohno K, Saitoh N, Nomura T, Nagamine S, Hishida H, Takahashi Y, Uruga T (2007). Bioreductive deposition of platinum nanoparticles on the bacterium *Shewanella algae*. Journal of Biotechnology.

[CR13] Kowalska J, Huszał S, Sawicki MG, Asztemborska M, Stryjewska E, Szalacha E, Golimowski J, Gawroński SW (2004). Voltammetric determination of platinum in plant material. Electroanalysis.

[CR14] Kowalska J, Asztemborska M, Bystrzejewska-Piotrowska G (2004). Platinum uptake by mustard *(Sinapis alba L.)* and maize *(Zea mays L.)*. Nukleonika.

[CR15] Lee W, An Y, Yoon H, Kweon H (2008). Toxicity and bioavailability of copper nanoparticles to the terrestrial plants mung bean (*Phaseolus radiatus*) and wheat (*Triticum awstivum*): plant uptake for water insoluble nanoparticles. Environmental Toxicology and Chemistry.

[CR16] Lin D, Xing B (2008). Root uptake and phytotoxicity of ZnO nanoparticles. Environmental Science and Technology.

[CR17] Lin, S., Reppert, J., Hu, Q., Hunson, J. S., Reid, M. L., & Ratnikova, T. (2009). Uptake, translocation and transmission of carbon nanomaterials in rice plants. *Small, 5*(10), 1128–1132. 10.1002/smll.200801556 19235197

[CR18] Luo X, Morrin A, Killard AJ, Smyth MR (2006). Application of nanoparticles in electrochemical sensors and biosensors. Electroanalysis.

[CR19] Maynard AD, Aitken RJ, Butz T, Colvin V, Donaldson K, Oberdorster G, Philbert MA, Ryan J, Seaton A, Stone V, Tinkle SS, Tran L, Walker NJ, Warheit DB (2006). Safe handling of nanotechnology. Nature.

[CR20] Mohanpuria P, Rana NK, Yadav SK (2008). Biosynthesis of nanoparticles: technological concepts and future applications. Journal of Nanoparticle Research.

[CR21] Oberdorster G, Maynard A, Donaldson K, Castranova V, Fitzpatrick J, Ausman K, Carter J, Karn B, Kreyling W, Lai D, Olin S, Monteiro-Riviere N, Warheit D, Yang H (2005). Principles for characterizing the potential human health effects from exposure to nanomaterials: elements of a screening strategy. Particle and Fibre Toxicology.

[CR22] Oughton DH, Hertel-Aas T, Pollicer E, Mendoza E, Joner EJ (2008). Neutron activation of engineered nanoparticles as a tool for tracing their environmental fate and uptake in organisms. Environmental Toxicology and Chemistry.

[CR23] Paz-Alberto AM, Sigua GC (2013). Phytoremediation: a green technology to remove environmental pollutants. American Journal of Climate Change.

[CR24] Rejeski D, Lekas D (2008). Nanotechnology field observations: scouting the new industrial west. Journal of Cleaner Production.

[CR25] Song JY, Kwon EY, Kim BS (2010). Biological synthesis of platinum nanoparticles using *Diospyros kaki* leaf extract. Bioprocess and Biosystems Engineering.

[CR26] Zhu H, Han J, Xiao JQ, Jin Y (2008). Uptake, translocation and accumulation of manufactured iron oxide nanoparticles by pumpkin plants. Journal of Environmental Monitoring.

